# Using multiple self-sorting for switching functions in discrete multicomponent systems

**DOI:** 10.3762/bjoc.16.233

**Published:** 2020-11-20

**Authors:** Amit Ghosh, Michael Schmittel

**Affiliations:** 1 Center of Micro and Nanochemistry and Engineering, Organische Chemie I, Universität Siegen, Adolf-Reichwein-Str. 2, D-57068 Siegen, Germany

**Keywords:** copper, fluorescence, self-assembly, self-sorting, zinc porphyrin

## Abstract

Over years self-sorting has developed into a powerful tool in supramolecular chemistry, for instance, to promote the error-free formation of intricate multicomponent assemblies. However, in order to use the enormous potential of self-sorting for sophisticated information processing more recent developments have focused on the reversible reconfiguration of multicomponent systems driven by multiple self-sorting protocols. The present mini review will provide an overview over the latest advancements in this field with a focus on reversibly switchable functions in discrete supramolecular systems.

## Introduction

Since self-sorting is meant to guide a directionless ensemble of molecular species toward a defined assortment of aggregates, the associated recognition processes are increasingly exploited for information handling. In nature, a high degree of self-sorting frequently constitutes the basis for regulating intricate functions, for instance, to control the biological processes that eventually sustain life on our planet [1]. A well-known example is the storage of an immense amount of information in the DNA, using the algorithm of base pairing (AT and GC) between the heterocycles adenine (A), thymine (T), guanine (G) and cytosine (C) [2]. Similarly, proteins, like microtubules and actin filaments, are self-sorted on the molecular level in living cells [3]. Furthermore, the smaller molecules of life such as sugars [4], peptides, and fatty acids [5] undergo self-sorting in the construction of a cell [6–7].

The above biological examples [2–3,6–7], convincingly illustrate that Nature utilizes self-sorting in fundamental biological processes. In contrast to sophisticated biological information processing, the majority of synthetic self-assembled systems so far is based on rather primitive versions of self-sorting using undemanding building blocks. The plethora of self-sorting systems depends on either the geometric fit of their global shapes and/or matching of their local interactions. Various noncovalent interactions, such as H‐bonding [8–9], metal–ligand coordination [10–13], electrostatic interactions [14], π‐stacking [15–16], dipole–dipole interactions [17] or hydrophobic interactions [18], have proven their significance as key players [19] in the creation of self-sorted supramolecular assemblies, such as 1D, 2D [20], and 3D architectures [21], polymers [22], gels [23], and most recently, of stand-alone devices [24] and molecular machines [25–27].

**Figure 1:**
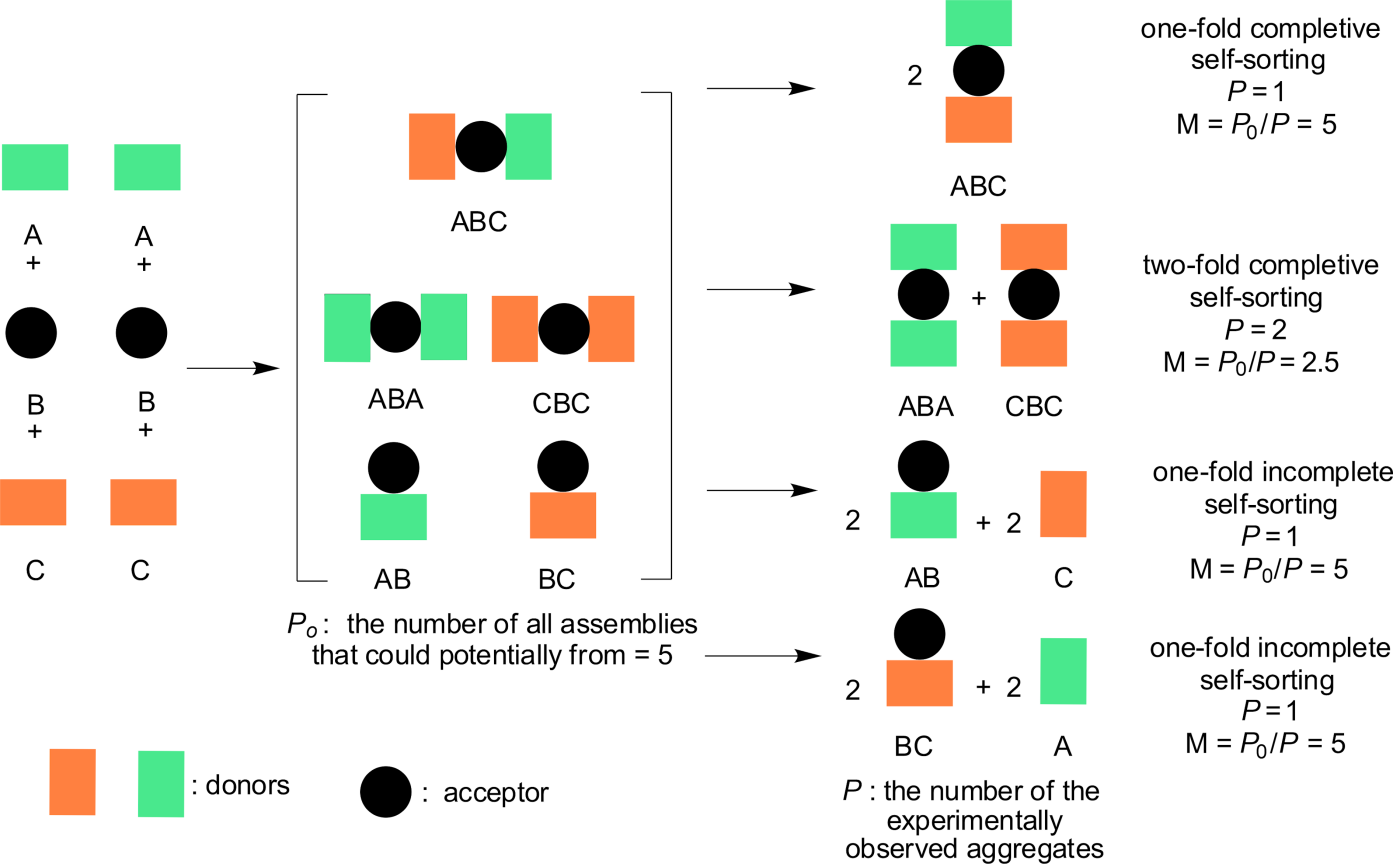
Some selected self-sorting outcomes and their qualitative and quantitative assessment.

On the discrete molecular level, self-sorting expresses the capacity to distinguish “self” from “non-self” in multicomponent mixtures [28]. Initially, the term self-sorting was only applied for the formation of well-defined homomeric assemblies (*narcissistic* self-sorting) but later it was expanded to the mutual recognition of different components (*social* self-sorting) [29]. In both cases the selection is based on the accurate read-out of specific information encoded in the molecules without any additional external help. Further definitions to describe self-sorting in qualitative and sometimes in quantitative terms have emerged later, such as the degree of self-sorting, the number of self-sorted species (*n*-fold), and the number of experimentally observed aggregates (*P*). The degree of self-sorting (*M*) was defined as *M* = *P*_0_/*P*, with *P*_0_ being the number of all assemblies that realistically may form (Figure 1).

**Figure 2:**
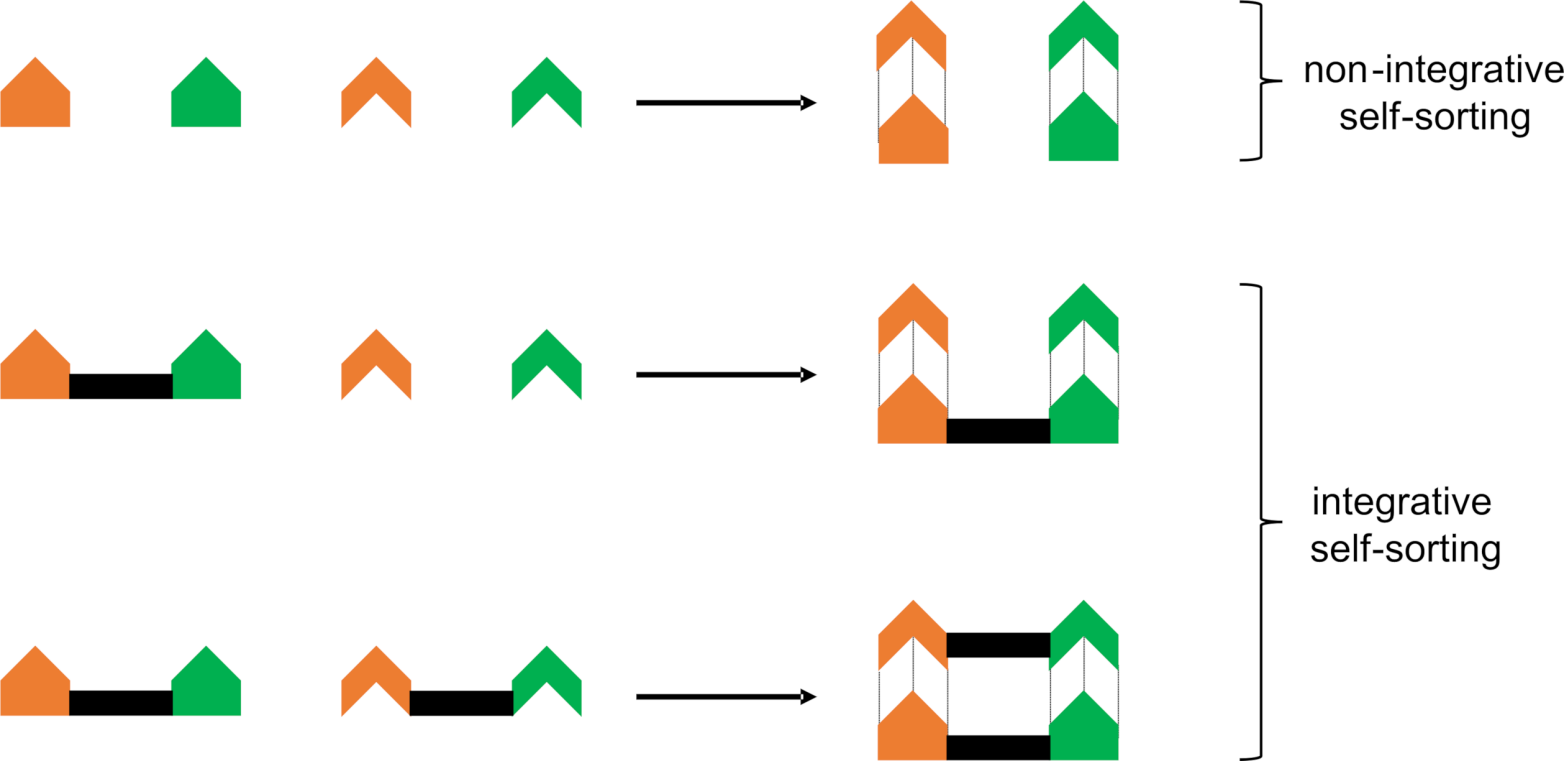
Illustration of an integrative vs a non-integrative self-sorting.

At present, simple qualitative descriptions are still dominating in the literature because they allow a quick, albeit imprecise, description of the self-sorting process. The most frequently used descriptors are *completive* vs *incomplete* self-sorting, and *integrative* vs *non-integrative* self-sorting. A completive self-sorting makes full use of all constituents in a given library, whereas an incomplete self-sorting describes mixtures containing one or several aggregates along with unused components. The integrative self-sorting [30] on the other hand denotes the formation of a single entity from all constituents using orthogonal binding motifs. In the self-sorting of more than two components, thus at least one ligand has to be multivalent (Figure 2).

Clearly, heteromeric self-sorting has a superior value for switching because it allows for several assortments and thus opens up a new arena called multiple self-sorting [31], for instance, upon the addition of an external input **C** to **AB**, when **B** in **AB** is replaced by the better binding **C** or **D**: **AB** → **AC**→ **AD**, a process that is of interest for setting up smart reaction networks.

For switching, the reconfigurable self-sorting is a protocol that Nature efficiently uses when responding to external stimuli [32]. The development of stimulus-responsive transformations between the supramolecular assemblies is a significant challenge in chemistry, in particular in regards to realizing biomimetic functions [33]. Over the years, a variety of chemical stimuli (pH [34], metal ions [35–37], ligands [38], solvents [39–41], and reagents [42]) have been successfully utilized in dual-state transformations. Multistate transformations with more than two well-defined states [43] should be beneficial for creating even smarter protocols, for instance, those controlling molecular machineries and complex logic gates. So far, the strategies with multiple self-sorting events have rarely been explored [44–46], because they need several precise transformations of supramolecular architectures, where each of the individual states should represent a thermodynamic minimum protected by a significant energy barrier. Overcoming the energy barrier may be solved by adding a stimulus for each self-sorting step.

Recently, excellent reviews have been reported by Nitschke et al*.*, covering the new aspects of supramolecule-to-supramolecule transformations, involving functions such as chemical purification, controllable guest uptake and release, and reagent storage along with catalysis [47]. However, neither of the examples in this compilation proved to be reversible. In the present minireview, we highlight the systematic indexing of examples as single, double, and multiple self-sorted systems and an evaluation of the functions during the reversible switching.

## Review

### Single self-sorting

The first chapter is not yet devoted to the multiple self-sorting events, but to the rare cases where a statistical ensemble of aggregates is converted to an *n*-fold completive mixture. Alike the material presented later we do not start from the ensemble of constituents, but already from aggregates.

**Figure 3:**
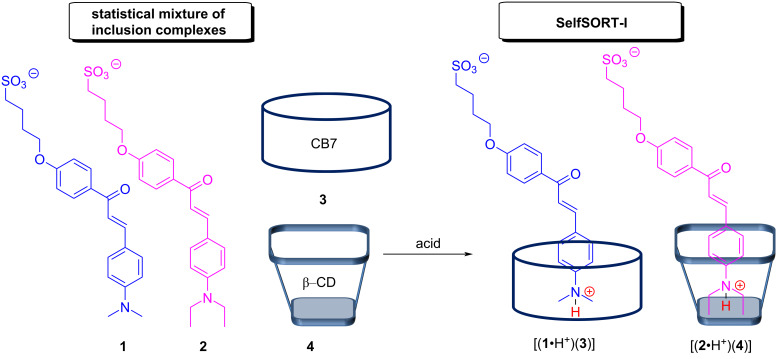
The pH-driven four-component 2-fold completive self-sorting based on host–guest chemistry.

Basilio and Parola demonstrated the pH-triggered 2-fold completive self-sorting of four components comprising β-cyclodextrin (**4**), cucurbit[7]uril (**3**), and the two chalcones **1** and **2** in aqueous solution [48]. When the hosts **3** and **4** were mixed with the *trans*-chalcones **1** and **2** as guests in a 1:1:1:1 ratio, instantly a statistical mixture formed displaying all four possible host–guest complexes. After the exposure to an acid, the ensuing rearrangement after the protonation of **1** and **2** furnished exclusively the two complexes [(**1**•H^+^)(**3**)] and [(**2**•H^+^)(**4**)] (Figure 3). The reduced affinity of the cucurbit[7]uril toward the protonated diethylamino-substituted guest in combination with the concomitant increased binding for the dimethylammonium derivative is the main reason behind this unusual social self-sorting phenomenon.

**Figure 4:**
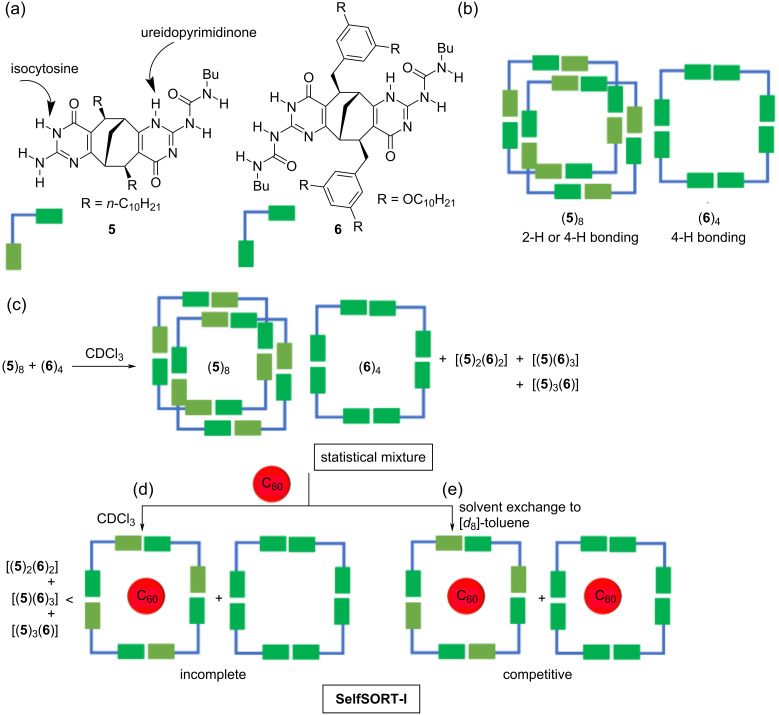
(a) The monomers **5** and **6** and their H-bonding array. (b) The hydrogen-bonded octameric and tetrameric tubes. (c) A representation of the complex mixture after combining the monomers **5** and **6** in CDCl_3_. (d) The partial separation of the mixture upon the selective C_60_ complexation by monomer **5**. The guest-induced rearrangement results in an incomplete self-sorted mixture. e) A solvent change from CDCl_3_ to [*d*_8_]-toluene leads to a 2-fold completive self-sorting upon treatment with C_60_.

Wärnmark and Orentas reported on a guest- and solvent-induced 2-fold self-sorting through hydrogen-bonding [49]. When the *C*_2_-symmetric monomers **5** and **6**, both exhibiting similar shapes except for different solubility-enhancing side chains (Figure 4a), were mixed in a 1:1 ratio in CDCl_3_, a complicated mixture was obtained (Figure 4c), mainly consisting of diverse hydrogen-bonded aggregates (Figure 4b). As revealed by NMR spectroscopy, the cyclic monomers **5** and **6**, each equipped with one Upy (ureidopyrimidinone) and one ICyt (isocytosine) moiety, furnished the very stable tetrameric cyclic aggregates by a cooperative 3H- and 4H‐bonding [50]. The addition of C_60_ to the mixture resulted in an incomplete self-sorting (Figure 4d) showing the complex [(**5**)_4_(C_60_)] and free (**6**)_4_ as the major species together with some mixed aggregates. Interestingly, the fullerene C_60_ was not taken up as a guest by the tetramer (**6**)_4_ in chlorinated solvents. For a more defined self-sorting, the authors switched the solvent from CDCl_3_ to [*d*_8_]-toluene. Now, a 2-fold completive self-sorting delivered the homoleptic inclusion complexes [(**5**)_4_(C_60_)] and [(**6**)_4_(C_60_)] (Figure 4e).

**Figure 5:**
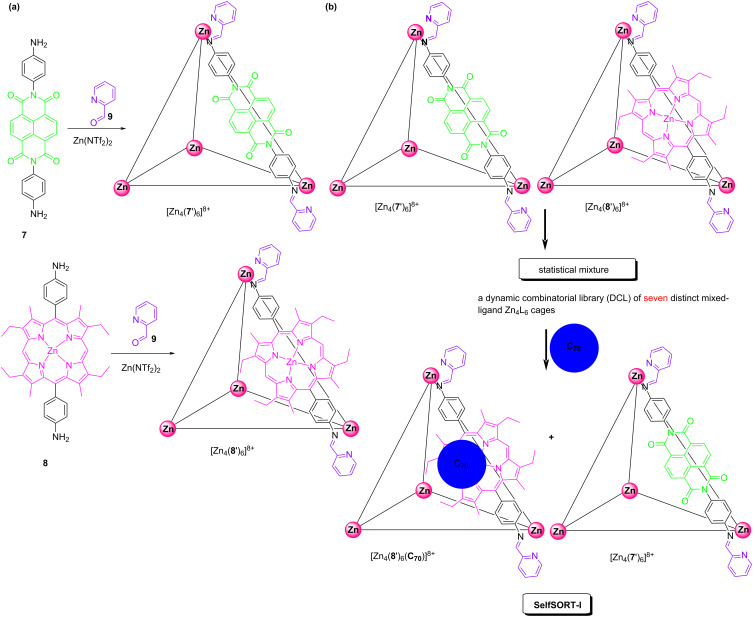
(a) Two new Zn_4_L_6_-type cages. (b) The encapsulation of C_70_ induced distinct reconstitutions within a dynamic library of mixed ligand Zn_4_L_6_ cages.

Equally, Schalley and Nitschke developed a guest-induced self-sorting based on two new Zn_4_L_6_ cages (Figure 5a) using the aldehyde **9** and the diamine subcomponents **7** and **8** that contained either the naphthalene diimide or zinc porphyrin moiety [51]. Both cages respond selectively to distinct chemical stimuli yielding different supramolecular products. The porphyrin ligands of the cage [Zn_4_(**8'**)_6_]^8+^ interacted favorably with C_70_ as a guest, whereas an electron-rich aromatic crown-ether did thread onto the electron-deficient naphthalene diimides of cage [Zn_4_(**7'**)_6_]^8+^ forming mechanically-interlocked catenanes. When both cages were mixed without C_70_, the dynamic combinatorial library (DCL) of seven compositionally distinct mixed-ligand Zn_4_L_6_ cages was observed (Figure 5b). An efficient self-sorting was only observed after the addition of the guest C_70_. As expected, the cage [Zn_4_(**8'**)_6_]^8+^ encapsulated the C_70_ (state SelfSORT-I), and forced the mixture to reconstitute into the 2-fold self-sorted homoleptic structures (Figure 5b).

**Figure 6:**
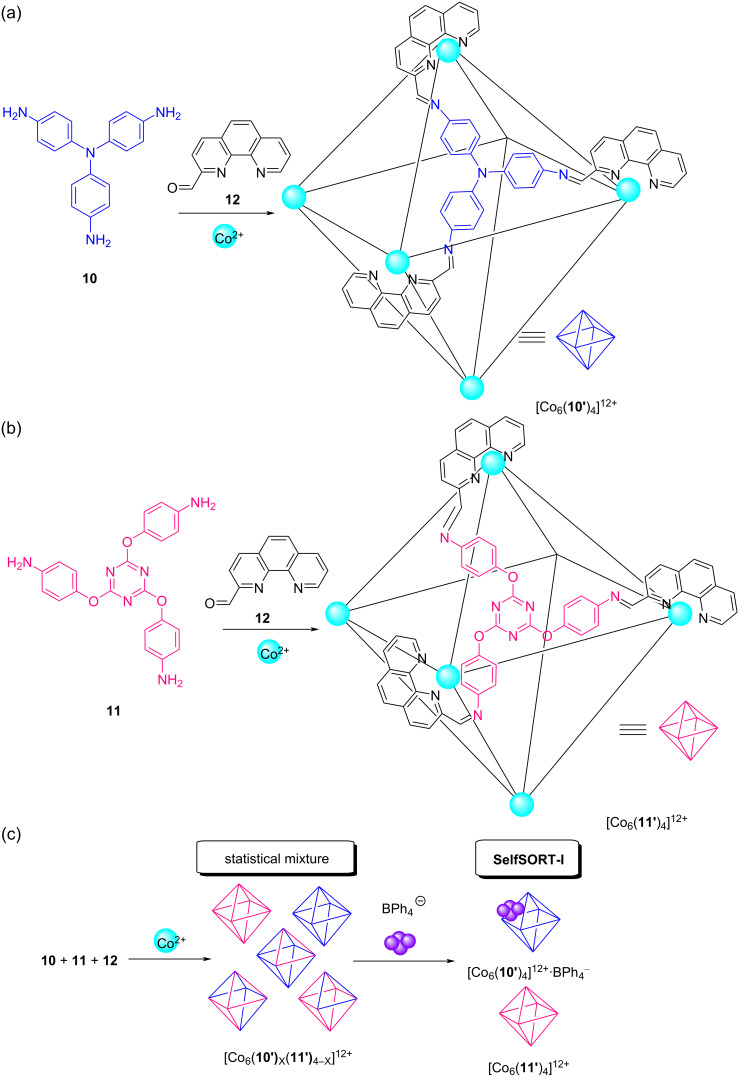
The formation of octahedral cages (a) [Co_6_(**10'**)_4_]^12+^ and (b) [Co_6_(**11'**)_4_]^12+^. (c) The 2-fold completive self-sorting after the addition of peripherally binding BPh_4_^−^.

In a separate work, Nitschke and co-workers reported on a series of homoleptic supramolecular M^II^_6_L_4_ pseudooctahedra (Figure 6a,b) that had formed from the subcomponent self-assembly of the triamines **10** and **11** in the presence of the aldehyde **12** and cobalt(II) ions [52]. When all components were mixed in a single reaction vessel, the ^1^H NMR spectrum indicated the formation of homoleptic as well as heteroleptic species. In the following, they explored the ability of the anions to amplify homoleptic cages by driving a 2-fold narcissistic self-sorting. When an excess amount of BPh_4_^–^ was added, NMR and ESIMS peaks indicated only formation of the homoleptic species (Figure 6c). The amplification to homoleptic species is realized through the peripheral binding of the anion BPh_4_^−^ at the cage [Co_6_(**10'**)_4_]^12+^ (SelfSORT-I).

### Double self-sorting (only structural)

**Figure 7:**
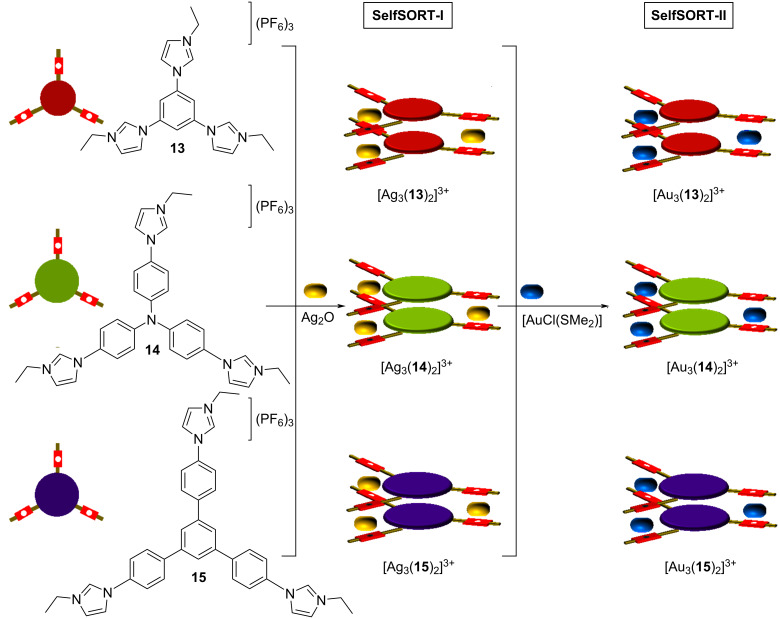
Exchange of Ag^+^ for Au^+^ ions in poly-NHC ligand-based organometallic assemblies.

Hahn et al. demonstrated for the first time that poly-NHC ligands furnish metallosupramolecular assemblies through narcissistic self-sorting [53]. The one-pot reaction of the tris-NHC ligands **13**–**15** with different backbones in the presence of Ag_2_O provided exclusively the three homomeric cylinders [Ag_3_(**13**)_2_]^3+^, [Ag_3_(**14**)_2_]^3+^, and [Ag_3_(**15**)_2_]^3+^ (state: SelfSORT-I in Figure 7). Upon the addition of gold(I) ions, a one-pot transmetalation triggered an exchange of the Ag^+^ ions for Au^+^ in the tris-NHC ligand-based cylinders (SelfSORT-II). Such type of transmetalation in metal–NHC complexes with a retention of the individual homomeric supramolecular assemblies has not been reported in literature.

**Figure 8:**
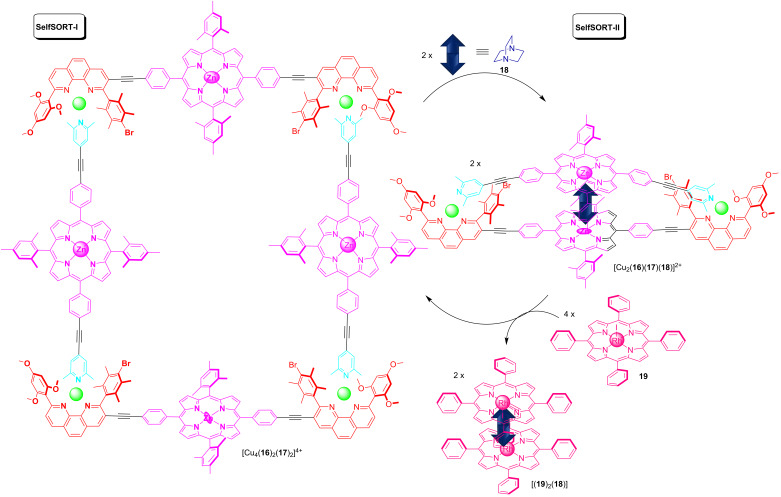
The reversible interconversion between the three-component rectangle [Cu_4_(**16**)_2_(**17**)_2_]^4+^ and the four-component sandwich complex [Cu_2_(**16**)(**17**)(**18**)]^2+^.

Recently, a quantitative and reversible structural interconversion of supramolecular structures was achieved by the inclusion and release of DABCO using a double self-sorting protocol [54]. Schmittel and co-workers reported on the three-component rectangle [Cu_4_(**16**)_2_(**17**)_2_]^4+^ (SelfSORT-I) that rearranged into the four-component sandwich complex [Cu_2_(**16**)(**17**)(**18**)]^2+^ (Figure 8) upon the addition of DABCO (SelfSORT-II). A full reversibility was achieved by the addition of the rhodium porphyrin **19** that reversed the system reviving the state SelfSORT-I. Since both self-sorted states exhibited a distinct fluorescence due to the changes at the zinc porphyrin sites, luminescence was used for the selective detection of DABCO in a mixture of various similar molecules.

**Figure 9:**
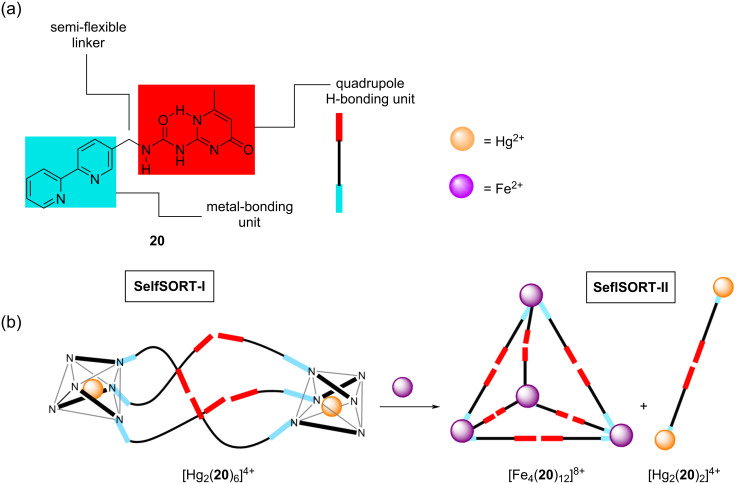
a) Chemical structure of the monomer **20** with its quadruple hydrogen-bonding array and a metal-affine 2,2’-bipyridine unit. b) Conversion of the helicate [Hg_2_(**20**)_6_]^4+^ to the *S*_4_-cage [Fe_4_(**20**)_12_]^8+^ and [Hg_2_(**20**)_2_]^4+^ based on double self-sorting.

Similarly, Shi controlled a conversion between helicates and a tetrahedral cage by varying the radius of the metal ion (Hg^2+^ vs Fe^2+^) [55]. They reported on the self-assembly of the monomer **20**, encompassing the quadruple DDAA hydrogen-bonding arrays and 2,2’-bipyridine units as the metal-coordination units (Figure 9a). When the ligand **20** was mixed with Fe^2+^ or Zn^2+^ ions, the tetrahedral cage complexes [M_4_(**20**)_12_]^8+^ were formed quantitatively. The flexibility through a methylene linker in **20** allowed the formation of the *S*_4_-symmetric cages [Fe_4_(**20**)_12_]^8+^ or [Zn_4_(**20**)_12_]^8+^. In contrast, metal ions (Hg^2+^) with a larger radius provided enough space for the hydrogen-bonding motif to set up different supramolecular architectures, such as the helicate [Hg_2_(**20**)_6_]^4+^ (SelfSORT-I). Furthermore, upon adding stoichiometric amounts of Fe(OTf)_2_ into the solution of the helicate, the conversion to the relatively stable *S*_4_-[Fe_4_(**20**)_12_]^8+^ was accomplished (state SelfSORT-II) (Figure 9b).

**Figure 10:**
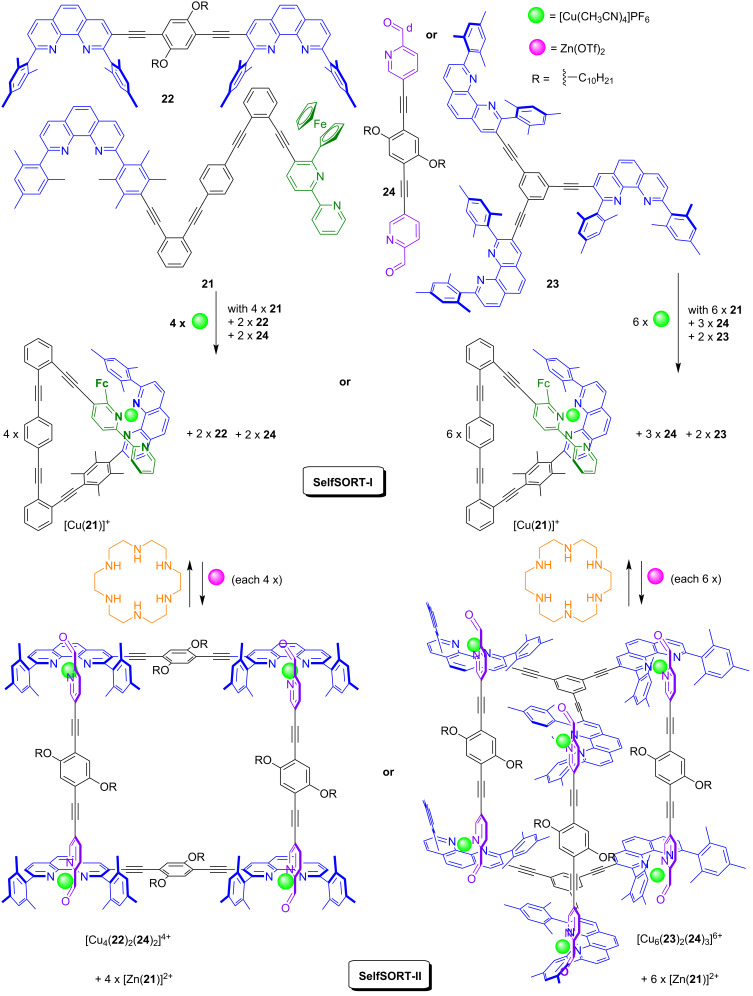
Communication between the nanoswitch **21** and the supramolecular assemblies [Cu_4_(**22**)_2_(**24**)_2_]^4+^ or [Cu_6_(**23**)_2_(**24**)_3_]^6+^ was guided by a double self-sorting.

In Figure 10, we present a multicomponent system where both, assembly and disassembly of either the supramolecular rectangle [Cu_4_(**22**)_2_(**24**)_2_]^4+^ or prism [Cu_6_(**23**)_2_(**24**)_3_]^6+^ was regulated by the nanoswitch **21** via metal ion translocation [56]. In detail, the addition of Zn(OTf)_2_ replaced copper(I) in nanoswitch [Cu(**21**)]^+^ with zinc(II), sending stoichiometric amounts of copper(I) ions as a second messenger to self-assemble the supramolecular rectangle or prism from ligands **22**–**24** (SelfSORT-II). Using hexacyclene, a strong complexation agent for zinc(II) ions, the communication was reversed and the supramolecular assemblies were disassembled to regain the initial state of the system (SelfSORT-I).

**Figure 11:**
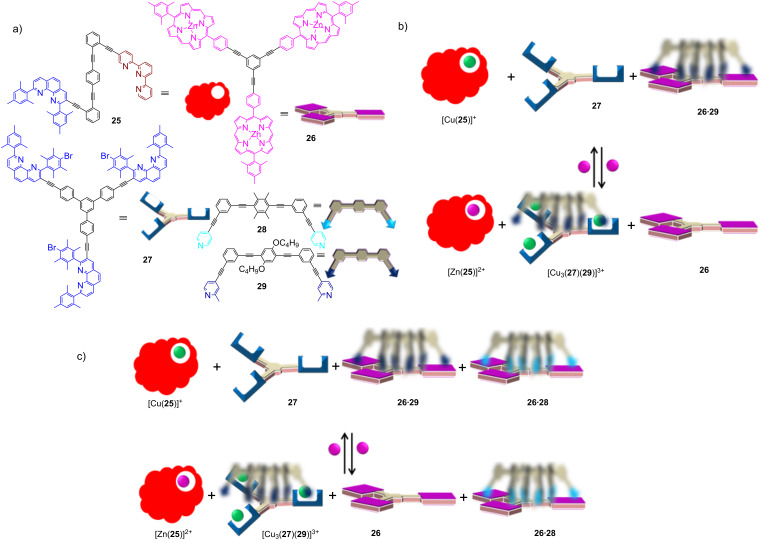
(a) The chemical structures and cartoon representations of the switch **25**, the decks **26** and **27**, and the bipeds **28** and **29**. (b) The double self-sorting led to a reversible interconversion of two different nanosliders triggered by the addition and removal of Zn^2+^. (c) Selective and reversible interconversion of nanosliders initiated by the addition and removal of Zn^2+^. Reproduced with permission from [57].

The use of multiple chemical inputs in combination with translocation is the key to remotely control the transformation of one nanodevice into another [57], for instance, in the reversible interconversion of nanosliders within six and seven-component networks. Upon the addition of three equiv of zinc(II) ions to three equiv of the nanoswitch [Cu(**25**)]^+^ (Figure 11b), the equivalent amount of copper(I) was released from [Cu(**25**)]^+^ and translocated to the free phenanthroline sites of the deck **27**. The ensuing complex [Cu_3_(**27**)]^3+^ now commanded the nanoslider **26**•**29** to dismantle and to transfer the biped **29** thus enabling the formation of the alternative device [Cu_3_(**27**)(**29**)]^3+^. In essence, a single input (Zn^2+^) was sufficient for the parallel disassembling and assembling of the nanodevices through the sequential two-component translocation. The potential of the networking system was further extended to a seven-component system, in which a selective translocation had to occur with one out of the two bipeds (Figure 11c).

### Double self-sorting (switching functions)

The superior value of double self-sorting strategies can be seen in the fact that novel switching functions are enabled in mixtures of several components (system chemistry).

**Figure 12:**
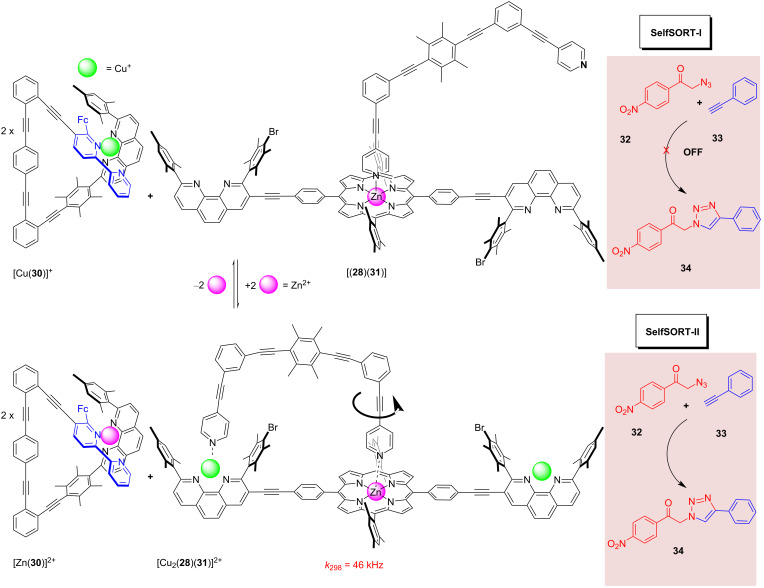
Double self-sorting leads to a catalytic machinery in SelfSORT-II, in which the 46 kHz-nanorotor acts as copper(I)-based catalyst in a click reaction.

A double self-sorting protocol in the mixture of the copper(I)-loaded nanoswitch [Cu(**30**)]^+^ and the pre-rotor complex [(**28**)(**31**)] was shown to generate a self-assembled catalytically active nanorotor upon the addition of zinc(II) ions [58]. In detail, the mixture of copper(I) ions and the ligands **28**, **30**, and **31** self-sorted into the copper(I)-loaded nanoswitch [Cu(**30**)]^+^ and the weakly bound pre-rotor assembly [(**28**)(**31**)] (state SelfSORT-I; Figure 12). The addition of [Zn(OTf)_2_] as input initiated the second self-sorting by releasing the copper(I) ions from the nanoswitch [Cu(**30**)]^+^ that transformed into [Zn(**30**)]^2+^. The liberated copper(I) ions were translocated to the pre-rotor along with the concurrent generation of [Cu_2_(**28**)(**31**)]^2+^, with the latter complex representing a three-component nanorotor operating at 46 kHz at room temperature (state SelfSORT-II). In [Cu_2_(**28**)(**31**)]^2+^ the rotator **28** exchanged rapidly between the two peripheral copper(I) phenanthroline sites of **31**. Importantly, the two self-sorting protocols also cleanly happened in the presence of the substrates **32** and **33**. However, now in state SelfSORT-II, the nanorotor [Cu_2_(**31**)(**28**)]^2+^ acted as a copper(I)-based catalyst for the click reaction of **32** and **33** affording the product **34**, whereas in state SelfSORT-I, no transformation **32** + **33** → **34** was observed, because the copper(I) is deeply embedded in [Cu(**30**)]^+^. This double self-sorting showed a full reversibility upon the addition and removal of zinc(II) ions along with ON/OFF catalytic behavior and reproducible yields of the product **34** (36%) in two subsequent cycles.

**Figure 13:**
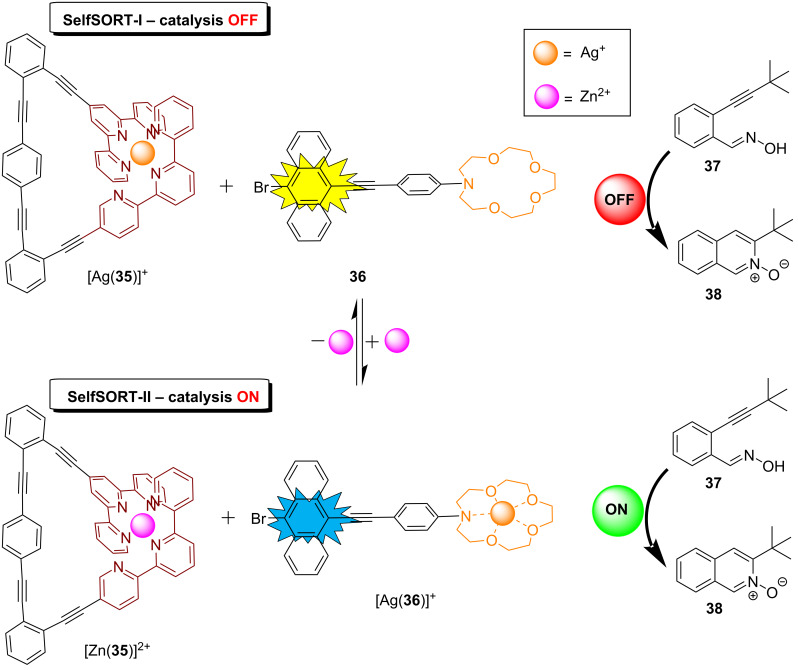
ON/OFF control of a networked catalytic catch–release system.

An innovative catch–release system with multiple functions combined the ON/OFF-adjustment of silver(I) catalysis and fluorescence monitoring [59]. Actually, the ratiometric luminescence response allowed the exact monitoring of the catalytic activity. In the initial incomplete self-sorting (state SelfSORT-I), the silver(I) ions were tightly captured within the cavity of the triangular nanoswitch **35** (“catch”) while the luminophore **36** was left uncoordinated exhibiting emission at 554 nm (Figure 13). Due to the firm complexation of the silver(I) ions in [Ag(**35**)]^+^ any catalysis was switched OFF. Upon the addition of zinc(II), the silver(I) ions were translocated as a second messenger from the nanoswitch [Ag(**35**)]^+^ to the anthracene-appended crown ether **36** in a 2-fold completive self-sorting, i.e., furnishing [Zn(**35**)]^2+^ and [Ag(**36**)]^+^ (state SelfSORT-II). In this state, SelfSORT-II, the emission emerged at 472 nm. If the state SelfSORT-II was generated in the presence of substrate **37** a cyclization to product **38** was seen (45% yield). In contrast, the silver(I) ions in [Ag(**35**)]^+^ (state: SelfSORT-I) were not able to act as a catalyst for the cyclization reaction. The double self-sorting along with ON/OFF catalytic behavior showed a full reversibility up to three cycles and provided 45%, 43%, and 41% yield, respectively, in subsequent cycles. The small decrease of the yield over three release/capture cycles was explained by a minor degradation of silver(I), a phenomenon equally seen in the emission channel.

**Figure 14:**
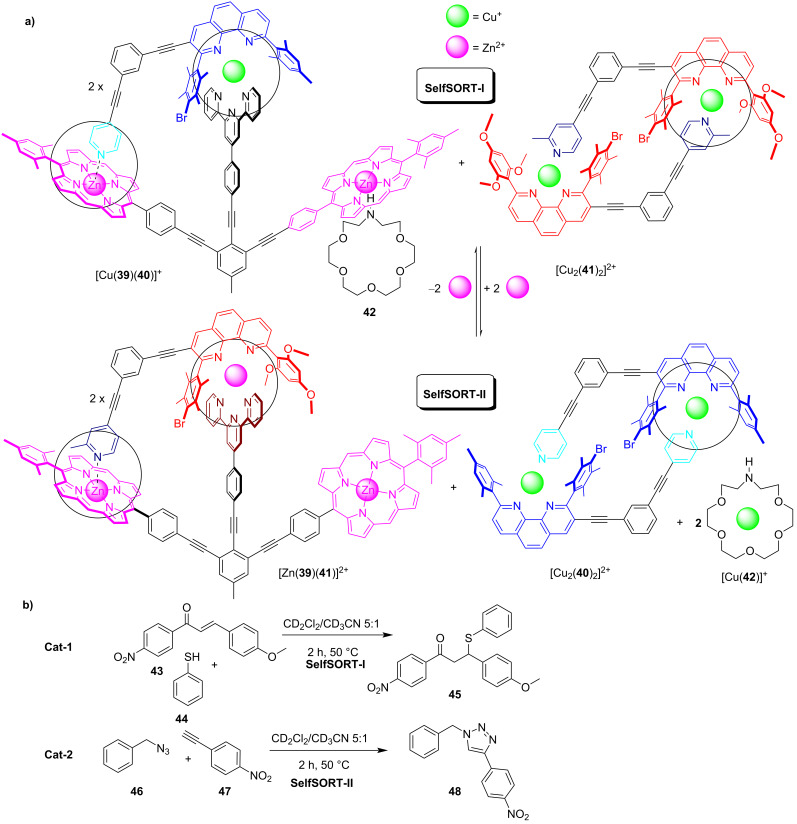
A multicomponent information system for the reversible reconfiguration of switchable dual catalysis.

An astounding modus operandi of a switchable catalytic system was realized based on information processing. The switchable system actually did not rely on a molecular switch in different toggling states, but on a smart seven-component mixture that reversibly regulated two diverse catalytic ON/OFF reactions in a double self-sorting protocol [60]. The operation of the network required the addition and removal of zinc(II) ions which triggered three distinct events in parallel: i) a mutually dependent self-sorting of different nanorotors and reshuffling of the components, ii) a switching between vastly different rotational exchange rates in the nanorotors that directly influenced catalysis, and iii) a toggling between two completely different catalytic processes. The main issue in the toggling process was to have two components transferred back and forth between two states although only one input was added from the outside. In order to achieve the two-component reshuffling, a component reservoir was needed aside of the nanorotor assembly. In the initial state SelfSORT-I, the nanorotor [Cu(**39**)(**40**)]^+^ was paired with [Cu_2_(**41**)_2_]^2+^, the latter being a reservoir for the rotator arm **41**. The addition of zinc(II) ions induced then the self-sorting 2 × [Cu(**39**)(**40**)]^+^ + [Cu_2_(**41**)_2_]^2+^ + 2 × Zn^2+^ (= SelfSORT-I) → 2 × [Zn(**39**)(**41**)]^2+^ + [Cu_2_(**40**)_2_]^2+^ + 2 × Cu^+^ (= SelfSORT-II) involving the transfer of two components, i.e., zinc(II) and **41**, to produce the zinc(II)-based rotor [Zn(**39**)(**41**)]^2+^ (Figure 14a) along with [Cu_2_(**40**)_2_]^2+^, representing a reservoir for the rotator **40**. As the liberated copper(I) ions proved to be unstable, 1-aza-18-crown-6 (**42**, 2.0 equiv) was added as a receptor, resulting in the overall transformation: 2 × [Cu(**39**)(**40**)(**42**)]^+^ + [Cu_2_(**41**)_2_]^2+^ + 2 × Zn^2+^ (= SelfSORT-I) → 2 × [Zn(**39**)(**41**)]^2+^ + [Cu_2_(**40**)_2_]^2+^ + 2 × [Cu(**42**)]^+^ (= SelfSORT-II).

With SelfSORT-I containing the heterocycle **42**, a known organocatalyst, and SelfSORT-II holding the potential click catalyst [Cu(**42**)]^+^, it was speculated that both networked states could be catalytically active thus allowing the ON/OFF regulation of a dual catalysis. For the evaluation of this property, the state SelfSORT-I was heated at 50 °C with 1.0 equiv of the catalyst **42** (with respect to the rotor), and 10.0 equiv (with respect to the rotor) of substrates **43**, **44**, **46**, and **47** in CD_2_Cl_2_/CD_3_CN 5:1. After heating the mixture for 2 h, 30% of the product **45** but no click product **48** was observed (Figure 14b). The addition of 1.0 equiv of zinc(II) ions (with respect to the rotor) generated the state SelfSORT-II. After heating this mixture using the identical conditions, 55% of the click product **48** was detected but no further conversion of the product **45**. Two consecutive catalytic cycles were run which displayed a remarkable reproducibility of the yields.

**Figure 15:**
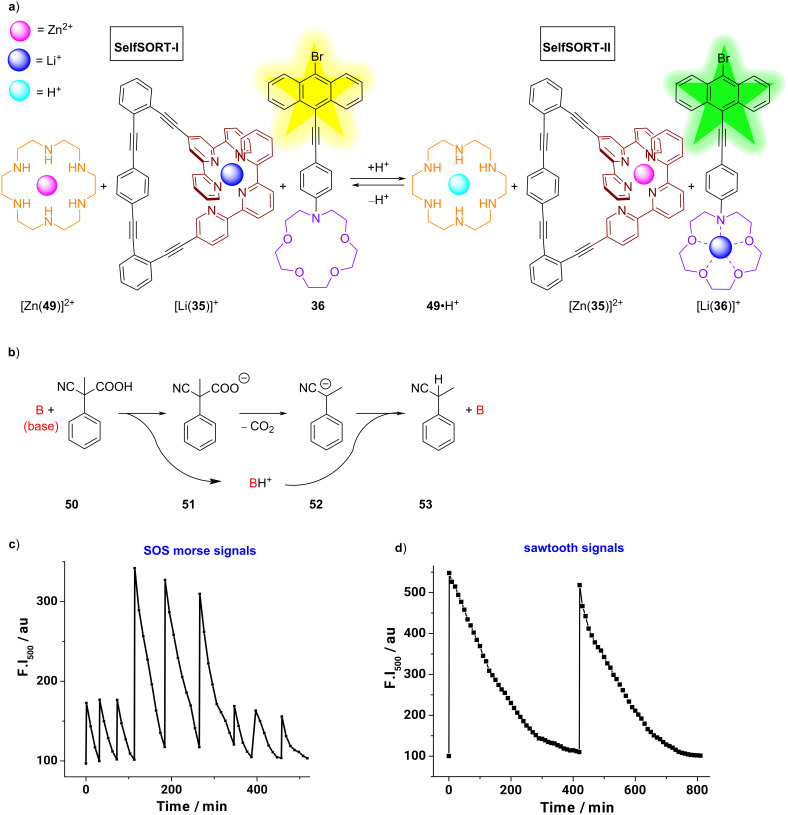
a) The chemically fueled cascaded ion translocation, monitored by distinct emission colors. b) Working principle of the chemical fuel **50**. Off-equilibrium lithium pulses generate c) SOS morse signals, and d) sawtooth signals. Adapted with permission from (Ghosh, A.; Paul, I.; Schmittel, M. *J. Am. Chem. Soc. ***2019,** *141,* 18954–18957 [61]). Copyright (2019) American Chemical Society.

Up to now, handling the time domain of ion translocation did not play a role in artificial molecular networking. The next example demonstrates a fully reversible and cascaded signaling system allowing the generation of lithium(I) pulses that, using a chemical fuel, introduce the time domain in the operation of an AND gate and thus in the field of (supra)molecular logic [61]. Based on the experience in stoichiometric metal–ligand self-sorting, the Schmittel group designed a two-step cascaded metal-ion translocation scheme using five components: hexacyclen (**49**), nanoswitch **35**, luminophore **36**, zinc(II) ions, and lithium(I) ions in a 1:1:1:1:1 ratio (Figure 15). In this small collection, the initial networked state SelfSORT-I was defined by a clean self-sorting of the Zn^2+^ ions within the cavity of hexacyclen (**49**), and of the Li^+^ ions inside the triangular nanoswitch **35** while the lithium-sensitive luminophore **36** was left unloaded (Figure 15a). In the following, the addition of TFA initiated a second self-sorting. It was shown that the acid protonated the ligand **49**, expelling zinc(II) from the complex [Zn(**49**)]^2+^. The liberated zinc(II) ions replaced the Li^+^ ions in the nanoswitch [Li(**35**)]^+^ and translocated them onto the luminophore **36**, thus finally generating the state SelfSORT-II composed of **49**•H^+^, [Zn(**35**)]^2+^, and [Li(**36**)]^+^. In the return process, the deprotonation of **49**•H^+^ by DBU triggered the back cascade translocation. Excitingly, when the self-sorted system in SelfSORT-I was treated with 2-cyano-2-phenylpropanoic acid (**50**) [62–63] as a chemical fuel, the protonation of **49** entailed the same cascade translocation resulting in SelfSORT-II but now with the effect that it slowly reversed back to the initial state (Figure 15b). Here, all translocations happen in an off-equilibrium system. Since the kinetic evolution of the lithium pulses was followed by color and luminescence changes of the lithium-sensitive probe **36**, the system is suited for a multitude of new applications, ranging from the generation of SOS morse signals to frequency-encoded AND gates (Figure 15c,d).

### Multiple self-sorting (without function)

**Figure 16:**
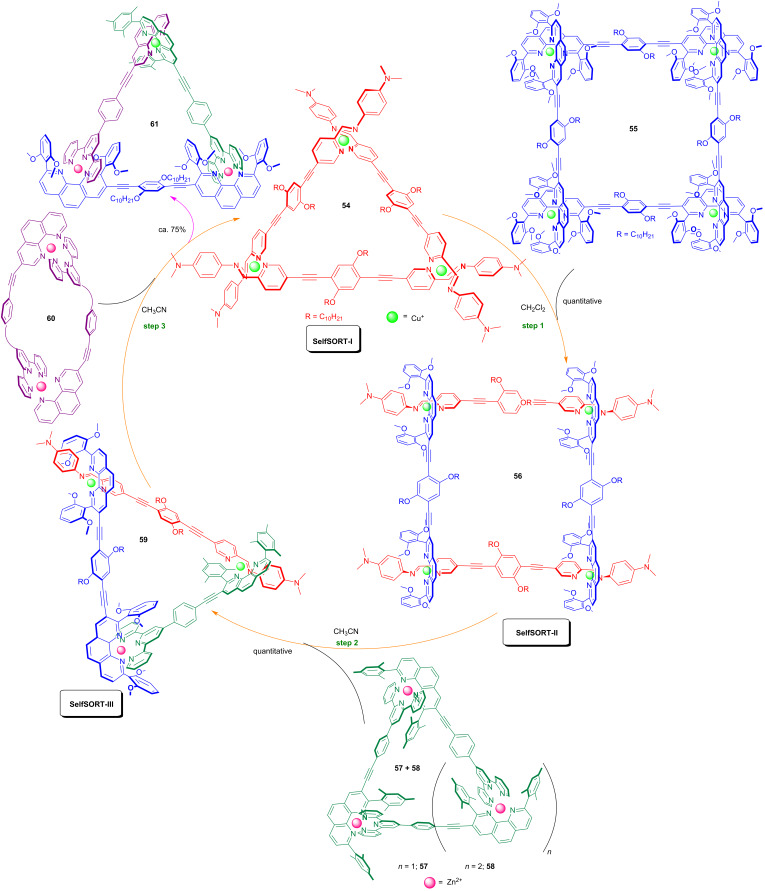
Cyclic metallosupramolecular transformations.

Using abiological self-assembled entities as chemical signals in completive self-sorting events, the Schmittel group demonstrated the cascaded metallosupramolecular transformation: SelfSORT-I → SelfSORT-II → SelfSORT-III → SelfSORT-I (Figure 16) [64]. In this unprecedented three-step reaction cycle that was designed on the corresponding association constants, the supramolecular two-component equilateral triangle **54** was selected as the starting entity in the state SelfSORT-I. The successive addition of the supramolecular architecture **55** and (**57** + **58**), a mixture of triangle and square, first induced a fusion into the three-component quadrilateral **56** (SelfSORT-II) and then to the five-component scalene triangle **59** (SelfSORT-III). The cycle was closed upon the addition of the supramolecular input **60** to the scalene triangle **59** which regenerated the equilateral triangle **54** (SelfSORT-I) along with the scalene triangle **61** as an output. As all self-sortings so far (SelfSORT-I → SelfSORT-II → SelfSORT-III) were thermodynamically downhill, a key element for achieving the last self-sorting was a strain release upon opening the dimeric species **60**.

**Figure 17:**
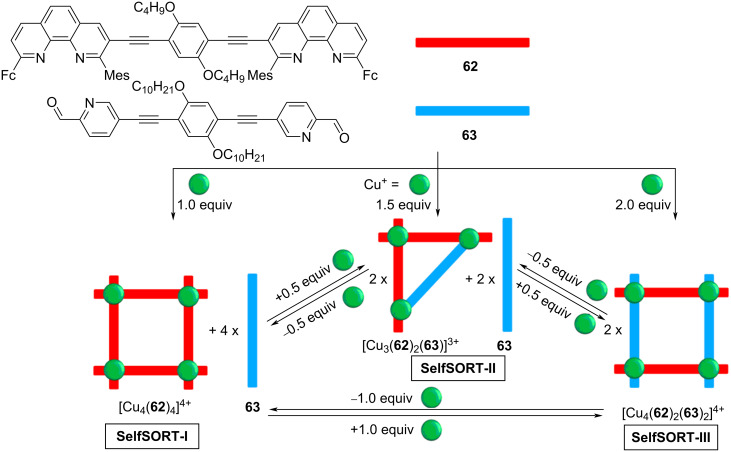
Fully reversible multiple-state rearrangement of metallosupramolecular architectures depending upon copper(I) stoichiometry. Reproduced from [65].

The multiple rearrangements as discussed so far are complex and fascinating but in most of the cases they represent irreversible transformations which prevent a use in reversible switching processes. A multistate reversible transformation requires a design with a cyclic interconversion of the involved supramolecular architectures. In this respect, the quantitative and reversible cyclic transformation of three metallosupramolecular architectures (Figure 17), i.e., square [Cu_4_(**62**)_4_]^4+^, triangle [Cu_3_(**62**)_2_(**63**)]^3+^, and rectangle [Cu_4_(**62**)_2_(**63**)_2_]^4+^ is a rare example [65]. The clean and quantitative (inter)conversion of one structure into another required the careful design of the ditopic ligands **62** and **63** and as metal ion Cu^+^ with a proper M:L ratio. At the time of publication, this cycle was the first example of a fully reversible three-state transformation of supramolecular architectures by varying the copper(I) stoichiometry [66]. Conceptually, the overall cyclic transformation discussed in Figure 17 was mainly driven by completive vs incomplete self-sorting protocols.

### Multiple self-sorting (with function)

**Figure 18:**
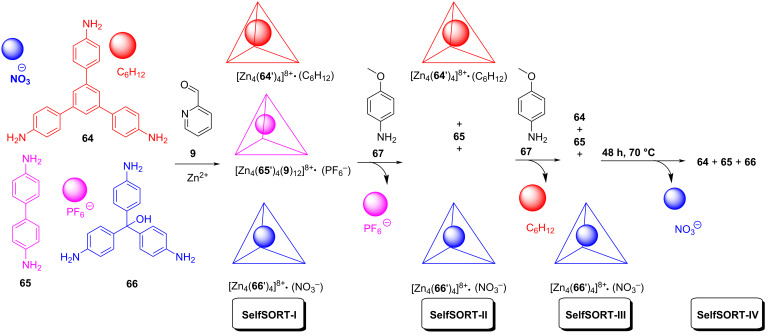
The selective encapsulation and sequential release of guests in a self-sorted mixture of three tetrahedral cages.

In the following work, Nitschke demonstrated a highly controlled sequential release of different guests using the same chemical signal but at a different stoichiometry [67]. A mixture of two triamines, **64** and **66**, the diamine **65**, 2-formylpyridine (**9**), and zinc(II) ions cleanly produced a self-sorted ensemble of three different tetrahedral cages through a 3-fold completive self-sorting (Figure 18). The cages are highly selective toward guest molecules, so that each did bind one of three guests, selectively and quantitatively. Each of the guests was sequentially released following the addition of 4-methoxyaniline (**67**), which reacted with the cages, disassembling each, and thus in turn promoting the release of the guest. The addition of 4 equiv of compound **67** to the state SelfSORT-I (= 1:1:1 mixture of the cages in the presence of the three guests C_6_H_12_, PF_6_^−^, and NO_3_^−^, respectively) at room temperature resulted after 30 min in the specific release of the guest (PF_6_^−^) with the consequent disassembly of the cage [Zn_4_(**65'**)_4_]^8+^ (SelfSORT-II). In SelfSORT-II, the host–guest complex [Zn_4_(**64'**)_4_]^8+^•(C_6_H_12_) remained intact, while ≈17% decrease in [Zn_4_(**66'**)_4_]^8+^•(NO_3_^−^) was observed. The addition of another aliquot of 4-methoxyaniline to SelfSORT-II disassembled the host–guest complex [Zn_4_(**64'**)_4_]^8+^•(C_6_H_12_) with the concomitant release of the encapsulated cyclohexane thus generating the state SelfSORT-III. In this process, the ^1^H NMR signals corresponding to [Zn_4_(**66'**)_4_]^8+^•(NO_3_^−^) remained unchanged. At last, heating the mixture to 70 °C for 48 h resulted in SelfSORT-IV, wherein the release of the remaining guest, NO_3_^−^, had happened due to the disassembly of the cage [Zn_4_(**66'**)_4_]^8+^, thus completing the sequential release of the guests. In the future, such a guest release could be used in signalling events. While being an impressive example, the sequential release is an irreversible process.

**Figure 19:**
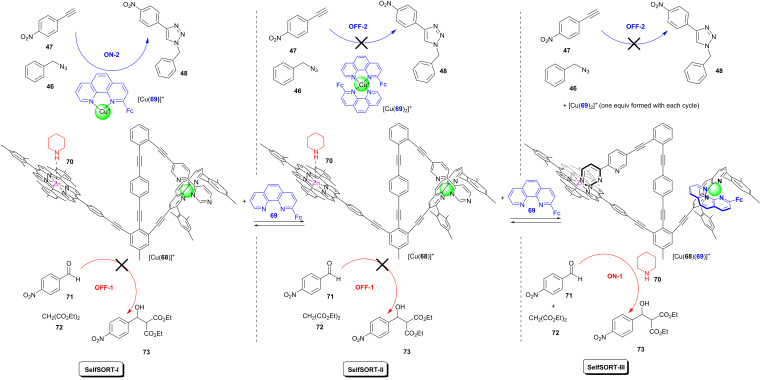
Two catalytic reactions are alternately controlled by a toggle nanoswitch.

The first multiply self-sorted catalytic machinery was demonstrated by the Schmittel group. It encompassed a three-state switching of a complex mixture with the triangular nanoswitch [Cu(**68**)]^+^ being a main player [68]. The reversible toggling between the states was accomplished by the addition of twice 2-ferrocenyl-1,10-phenanthroline (**69**) followed by the addition of copper(I) ions (Figure 19). In the state SelfSORT-I, representing an incomplete self-sorted mixture of ten components, piperidine (**70**) was firmly bound at the zinc porphyrin binding site to nanoswitch [Cu(**68**)]^+^ preventing its action as an organocatalyst (OFF-1), while the copper catalyst [Cu(**69**)]^+^ was available to catalyze a click reaction between 4-nitrophenylacetylene (**47**) and benzyl azide (**46**) (ON-2). The addition of 1 equiv of phenanthroline **69** to the state SelfSORT-I generated the catalytically dormant state SelfSORT-II (OFF-1, OFF-2), because [Cu(**69**)_2_]^+^ proved to be inactive as a catalyst. The further addition of one equivalent of **69** produced the state SelfSORT-III, in which phenanthroline **69** reacted with the intramolecular complex [Cu(**68**)]^+^ generating the intermolecular complex [Cu(**68**)(**69**)]^+^. As a result the toggling arm in [Cu(**68**)]^+^ had to move to the zinc porphyrin station affording [Cu(**68**)(**69**)]^+^. Whereas the organocatalyst **70** was firmly attached to the zinc porphyrin moiety of [Cu(**68**)]^+^, in [Cu(**68**)(**69**)]^+^ it was expelled into solution unfolding its catalytic activity in a Knoevenagel addition reaction. At the same time, the click reaction remained shut down (ON-1, OFF-2). In sum, the three interdependent states SelfSORT-I to III regulated two different reaction outcomes and an OFF state.

**Figure 20:**
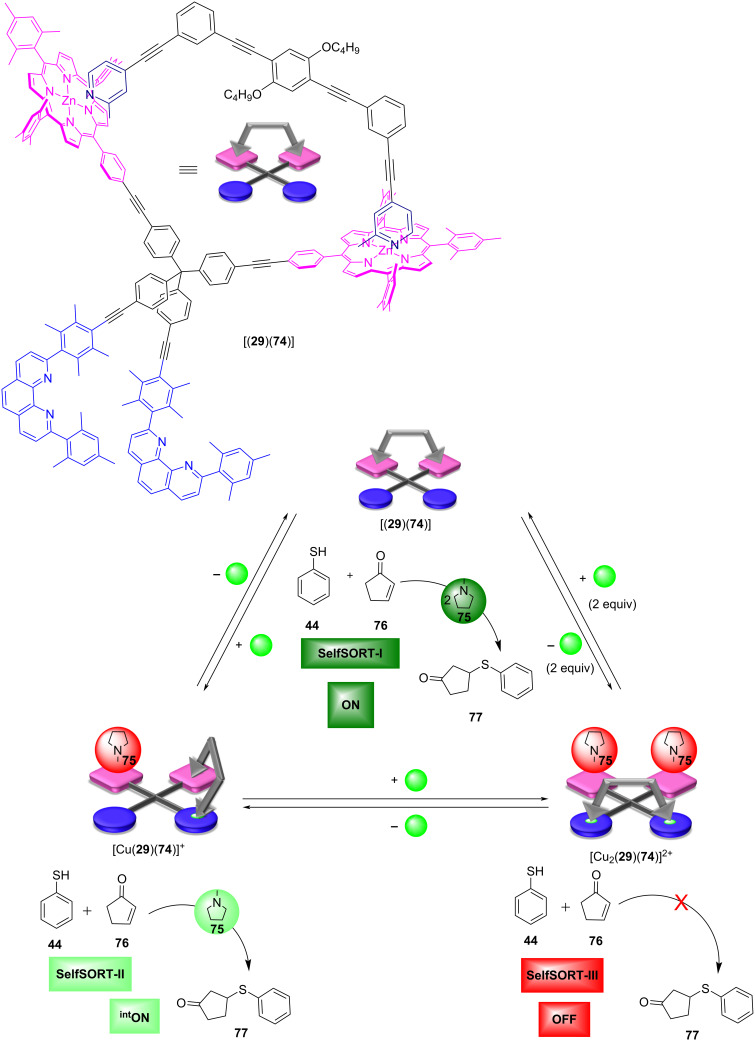
A biped walking along a tetrahedral track and unfolding its catalytic action. Adapted with permission from https://pubs.acs.org/doi/10.1021/acs.inorgchem.7b02703 [75]. Copyright (2018) American Chemical Society. Further permissions related to the material excerpted should be directed to the ACS.

In biology, motor proteins carry out essential tasks by walking along polymeric filaments [69–71]. In the last few years, biomolecular walkers have been an inspiration to develop a variety of artificial walkers that walk step-by-step along a track [72–74]. In this spirit, the Schmittel group developed a supramolecular walker consisting of the track **74** and the biped ligand **29** (1:1) [75]. Upon the addition of copper(I) ions the aggregate [(**29**)(**74**)] converted into [Cu_2_(**29**)(**74**)]^2+^ via the intermediate complex [Cu(**29**)(**74**)]^+^ (Figure 20).

Along this sequence, the two picoline feet of **29** walked from the ZnPor stations to the copper(I) phenanthroline stations of **74** through three self-sorting events (three-state switching). Finally, the consequences of forward and backward walking of the biped ligand **29** were studied in the presence of two equiv of *N*-methylpyrrolidine (**75**). The latter should be able to catalyze the conjugate addition of thiophenol (**44**) to 2-cyclopentenone (**76**) furnishing product **77**. In SelfSORT-I, 27% of the product **77** was afforded under standardized conditions, because both equiv of pyrrolidine **75** are free in solution. Upon the addition of one equiv of copper(I) ions, i.e., in SelfSORT-II, the yield of **77** increased by 12% to a total yield of 39%. This finding suggested that the catalytic activity in SelfSORT-II was reduced to roughly half of the initial activity in SelfSORT-I. Finally, the addition of two equiv of copper(I) ions generated SelfSORT-III where no additional product was afforded, indicating an OFF state of the catalytic machinery. To reverse the system, two equiv of 2-ferrocenyl-9-mesityl-1,10-phenanthroline were added as well as consumed amounts of substrates **44** and **76**. After walking back from SelfSORT-III to SelfSORT-II, the product yield increased by 12% and in SelfSORT-I by 24%, demonstrating the reversibility of walking and catalytic activity. In sum, this study demonstrated partial and full release/binding of the organocatalyst **75** during the walking of the biped.

**Figure 21:**
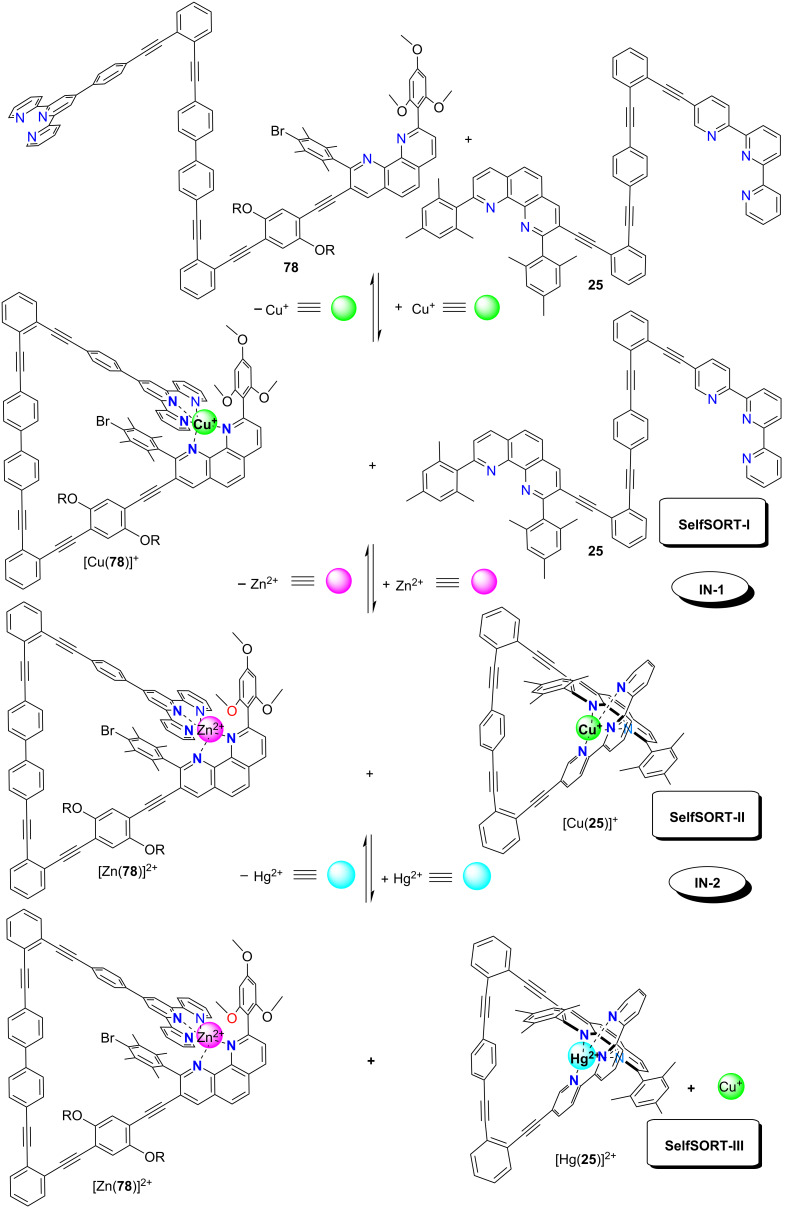
A three state supramolecular AND logic gate.

Rather recently, the Schmittel group described a precise intermolecular communication system, in which multiple self-sorting steps set up a catalytic AND gate mimicking the concatenation of biological information relays in activating enzymatic activity (Figure 21) [76]. In detail, the work involved the proper handling and networking of twelve components (two distinct nanoswitches **25** and **78**; Zn^2+^, Hg^2+^, Cu^+^ metal ions; stator **79**, rotator **80** and DABCO (**18**) as dynamic axle of the rotor assembly; hexacyclen to selectively remove the metal ions for regaining original states; two reactants and the product of the click reaction), requiring a systems chemistry approach [77]. At the heart of the logic operation, the two nanoswitches **25** and [Cu(**78**)]^+^ acted as a networked ensemble AND gate, formed from **25**, **78**, and Cu^+^ (1:1:1) in a clean incomplete self-sorting process. The AND gate itself was actuated by two metal-ion inputs (Zn^2+^ and Hg^2+^) generating a stoichiometric Cu^+^ output in the state SelfSORT-III (Figure 21).

**Figure 22:**
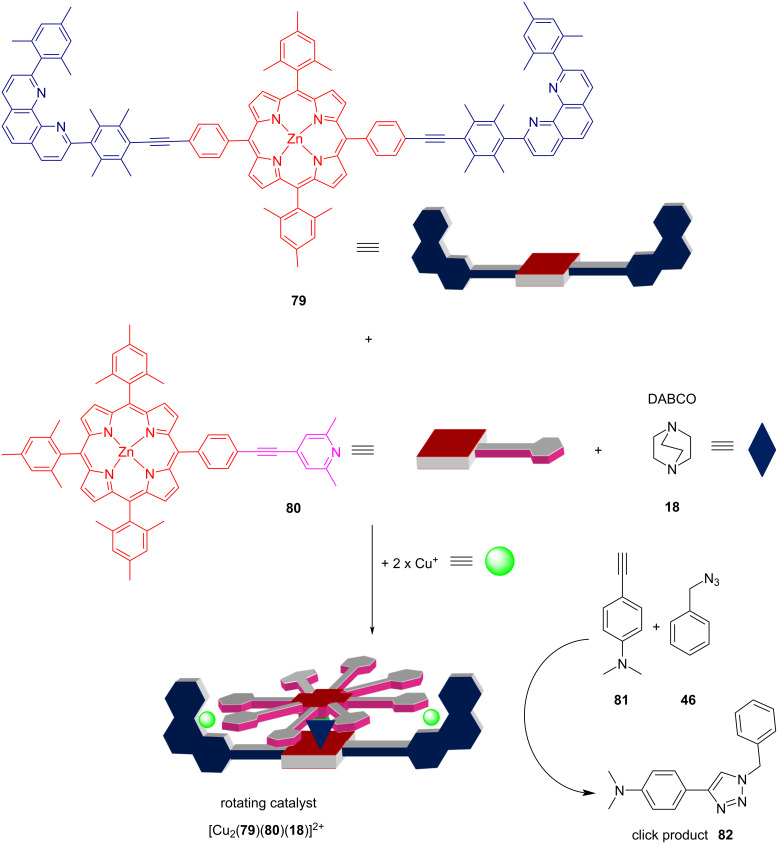
Four-component nanorotor and its catalytic activity. Adapted with permission from (Biswas, P. K.; Saha, S.; Gaikwad, S.; Schmittel, M. *J. Am. Chem. Soc.* **2020,** *142,* 7889–7897 [77]). Copyright (2020) American Chemical Society.

The liberated copper(I) ions were used to self-assemble the four-component rotor [Cu_2_(**79**)(**80**)(**18**)]^2+^ that itself triggered the catalysis of a click reaction (**81** + **46** → **82**) as shown in Figure 22. In conclusion, the output of the AND gate regulated both the assembly of the multicomponent machinery and the catalytic output.

## Conclusion

In conclusion, the collected examples convincingly demonstrate the power of self-sorting for achieving and switching functions in a systems chemistry approach. Most notably, the reproducibility of reconfiguring these multicomponent ensembles will encourage further work in improving information processing in smart mixtures.
